# A remarkable diversity of bone-eating worms (*Osedax*; Siboglinidae; Annelida)

**DOI:** 10.1186/1741-7007-7-74

**Published:** 2009-11-10

**Authors:** Robert C Vrijenhoek, Shannon B Johnson, Greg W Rouse

**Affiliations:** 1 Monterey Bay Aquarium Research Institute, Moss Landing, CA, USA; 2 Scripps Institution of Oceanography, UCSD, La Jolla, CA, USA

## Abstract

**Background:**

Bone-eating *Osedax *worms have proved to be surprisingly diverse and widespread. Including the initial description of this genus in 2004, five species that live at depths between 25 and 3,000 m in the eastern and western Pacific and in the north Atlantic have been named to date. Here, we provide molecular and morphological evidence for 12 additional evolutionary lineages from Monterey Bay, California. To assess their phylogenetic relationships and possible status as new undescribed species, we examined DNA sequences from two mitochondrial (*COI *and *16S *rRNA) and three nuclear genes (*H3*, *18S *and *28S *rRNA).

**Results:**

Phylogenetic analyses identified 17 distinct evolutionary lineages. Levels of sequence divergence among the undescribed lineages were similar to those found among the named species. The 17 lineages clustered into five well-supported clades that also differed for a number of key morphological traits. Attempts to determine the evolutionary age of *Osedax *depended on prior assumptions about nucleotide substitution rates. According to one scenario involving a molecular clock calibrated for shallow marine invertebrates, *Osedax *split from its siboglinid relatives about 45 million years ago when archeocete cetaceans first appeared and then diversified during the late Oligocene and early Miocene when toothed and baleen whales appeared. Alternatively, the use of a slower clock calibrated for deep-sea annelids suggested that *Osedax *split from its siboglinid relatives during the Cretaceous and began to diversify during the Early Paleocene, at least 20 million years before the origin of large marine mammals.

**Conclusion:**

To help resolve uncertainties about the evolutionary age of *Osedax*, we suggest that the fossilized bones from Cretaceous marine reptiles and late Oligocene cetaceans be examined for possible trace fossils left by *Osedax *roots. Regardless of the outcome, the present molecular evidence for strong phylogenetic concordance across five separate genes suggests that the undescribed *Osedax *lineages comprise evolutionarily significant units that have been separate from one another for many millions of years. These data coupled with ongoing morphological analyses provide a solid foundation for their future descriptions as new species.

## Background

*Osedax*, a recently discovered genus of bone-eating marine worms, are proving to be far more diverse and geographically widespread than initially realized. The genus was described from two newly discovered species found on whalebones recovered from 2,893 m depth in Monterey Bay, California [1]. Subsequently, three additional species were described from depths between 30 and 3,000 m in the Atlantic and Pacific oceans [2-4]. Now, five additional distinct evolutionary lineages are recognized from Monterey Bay, but these putative species remain to be formally described [5-8]. Here we report genetic evidence for seven additional putative species. Given this unexpected diversity of *Osedax *worms with distinct morphologies, depth ranges and ecological characteristics, a detailed examination of their evolutionary history is warranted.

The initial description of *Osedax *[1] included a phylogenetic analysis that placed the new genus in the polychaete annelid family Siboglinidae, which also includes the now obsolete tubeworm phyla Vestimentifera and Pogonophora [9,10]. As adults, all siboglinids lack a functional digestive system and rely entirely on endosymbiotic bacteria for their nutrition. The other siboglinid taxa host chemosynthetic bacteria and live in reducing marine environments such as hydrothermal vents, hydrocarbon seeps and anoxic basins. *Osedax*, however, are unique because they penetrate and digest bones with the aid of heterotrophic bacteriathat are housed in a complex branching *root *system [6,11]. *Osedax *also differ because they exhibit extreme sexual dimorphism involving dwarf (paedomorphic) males that live as *harems *within a female's tube [1,4,12].

**Figure 1:**
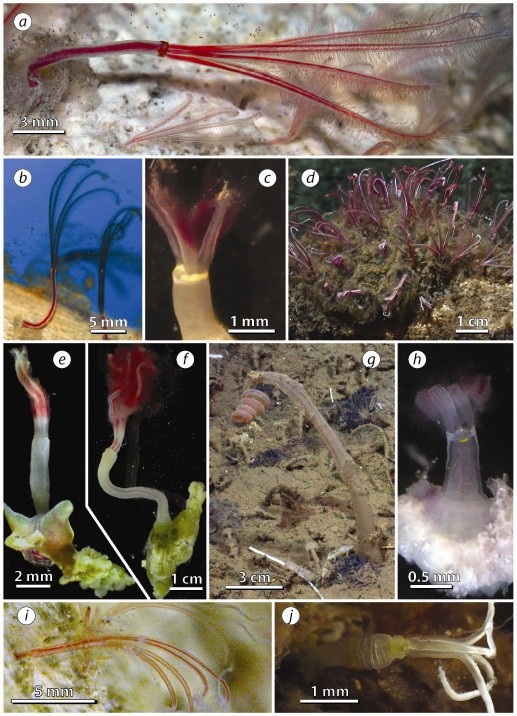
**Morphological diversity among *Osedax *lineages from Monterey Bay, CA**. Individual whale-falls are denoted by their depths in meters: (*a*) *O*. orange collar from whale-633; (*b*)*O*. yellow-collar from whale-385; (*c*)*O*. white-collar from whale-1018; (*d*)*O*. *frankpressi *from whale-2893; (*e*)*O*. *roseus *from whale-1018; (*f*)*O*. *rubiplumus *from whale-2893; (*g*)*O*. spiral from whale-2893; (*h*)*O*. yellow-patch from whale-1018; (*i*)*O*. nude-palp C from whale-1018; and (*j*)*O*. nude-palp D from whale-1820. Approximate scale bars are provided in each panel.

Considering DNA sequence divergence between the only two species known at the time, Rouse et al. [1] suggested that *Osedax *may have begun to diversify during the late Eocene, around 42 million years ago (MYA), perhaps coinciding with the origin of large oceanic cetaceans. Yet, this hypothesis must be reexamined in view of our current discoveries of far greater morphological and molecular diversity in the genus (Fig. 1). Our present goals were to better characterize the genetic differences among the five named species and to use this information as a foundation for clarifying evolutionary relationships among the 12 undescribed operational taxonomic units, OTUs (Table 1). We examined DNA sequences from five genes. Mitochondrial cytochrome-*c-*oxidase subunit 1 (*COI*) was used to assess levels of sequence diversity within and among all 17 OTUs and to provide DNA barcodes that would facilitate the identification of *Osedax *species in subsequent discoveries. Phylogenetic analyses were conducted independently with mitochondrial *COI *and *16S *rRNA and with three nuclear genes, Histone-*H3*, *18S *and *28S *rRNA. A combined analysis involving all five genes provided a robust phylogeny for the genus and identified several well-supported species-groups that diversified over a relatively short time scale, though the timing of these events during either the Mesozoic or Cenozoic remains uncertain. Formal descriptions of the new species from Monterey Bay are currently underway (Rouse, in progress).

**Table 1:** Characteristics of *Osedax *OTUs.

Taxa	Authority^1^	Depth (m)	Approx. Size^2^	Palp color	Pinnules^3^	Oviduct^4^	Roots	Museum Voucher^5^
*O. rubiplumus*	Rouse et al. 2004	1820--2893	59	brilliant red	out	long	long, branched	CASIZ 170238
*O. frankpressi*	Rouse et al. 2004	1820--2893	23	red with white lateral stripe	in	long	robust, lobate	CASIZ 170235
*O. mucofloris*	Glover et al. 2005	30--125	14	white to pink	in	long	robust, lobate	
*O. japonicus*	Fujikura et al. 2006	224--250	16	pink	out	short	robust, lobate	
*O. roseus*	Rouse et al. 2008	633--1820	24	bright red	out	long	long, branched	SIO-BIC A979-981
*O*. spiral	Braby et al. 2007	2893	25	no palps	none	absent	long thin filaments	SIO-BIC A1639
*O*. yellow-collar	Braby et al. 2007	383	18	red	both	long	robust, lobate	SIO-BIC A1640
*O*. orange-collar	Braby et al. 2007	383--1018	18	red	both	long	robust, lobate	SIO-BIC A1641
*O*. nude-palp-A	Jones et al. 2008	1820	25	red	none	?	?	SIO-BIC A1642
*O*. nude-palp-B	Jones et al. 2008	2893	25	red	none	?	?	SIO-BIC A1643
*O*. nude-palp-C	Rouse et al. 2009	1018	12	red	none	?	?	SIO-BIC A1644
*O*. nude-palp-D	this report	1018-1820	12	red	none	?	?	SIO-BIC A1645
*O*. nude-palp-E	this report	1018	12	red	none	?	robust, lobate	SIO-BIC A1646
*O*. nude-palp-F	this report	2892	18	red	none	?	robust, lobate	SIO-BIC A1647
*O*. white-collar	this report	1018	6	red with white stripe	both	long	robust, lobate	SIO-BIC A1648
*O*. yellow-patch	this report	633--1018	5	pale	both	long	robust, lobate	SIO-BIC A1649
*O*. green-palp	this report	1820	3	red/green	?	long	robust, lobate	SIO-BIC A1650
*S. brattstromi*	Webb 1964	600	40	red	none	absent	none	

^1^Authority and date of species description, or reference to first report in literature if species is undescribed.

^2^Maximum length of truck and crown (mm) when preserved.

^3^Orientation of pinnules if present: in = inward; out = outward; both = inward & outward; none = no pinnules.

^4^Extension of oviduct beyond trunk.

^5^Abbreviations: CASIZ, California Academy of Sciences Invertebrate Zoology collection; SIO-BIC, The Scripps Institutionof Oceanography Benthic Invertebrates Collection.

## Results

We examined DNA sequences from five genes (Table 2). Substitution models were estimated separately for each gene. The percentage GC content was lower in the mitochondrial genes (33.9 - 40.2%) than in the nuclear genes (47.8 - 50.2%). The two protein-coding genes, *COI *and *H3*, exhibited the highest sequence divergence. Ratios of transitions to transversions were nearly one, and ratios of synonymous to non-synonymous substitutions were comparable for *COI *and *H3*. For each gene, the sequences were partitioned by codon position, and substitution parameters were estimated separately for each position. Indels were found in all three of the rRNA genes. On average, the lengths of indels and numbers of distinct haplotypes were similar. Ratios of transitions to transversions were about one-half for the three ribosomal genes.

**Table 2:** Characterization of DNA sequences and the substitution models used to correct for saturation in the Bayesian analyses.

DNA	Length	Mean % divergence^1^	% GC content	Ts:Tv^2^			Model
Protein-coding					Synonomous^3^	Nonsynonymous	
						
mt *COI*	1005	17.0	41.0	1.304	259.42	94.58	GTR+SS
nuc *H*3	371	13.9	48.8	1.075	222.23	71.77	Sym+SS
Ribosomal					Indels^4^	Size^5^	
						
mt 16*S*	506	11.1	32.6	0.527	3	1.0	GTR+I+G
nuc 18*S*	1644	5.9	48.0	0.526	5	1.18	GTR
nuc 28*S*	400	8.6	47.4	0.584	4	1.14	GTR+I+G

^1 ^Uncorrected pairwise sequence divergence.

^2 ^Ratio of transitions to transversions.

^3 ^Protein coding genes: synonymous substitutions vs. non-synonymous.

^4 ^Ribosomal genes: the number of distinct haplotypes that differed for indels (insertion/deletion).

^5 ^Average length in base pairs of indels.

### Phylogenetic analyses

**Figure 2:**
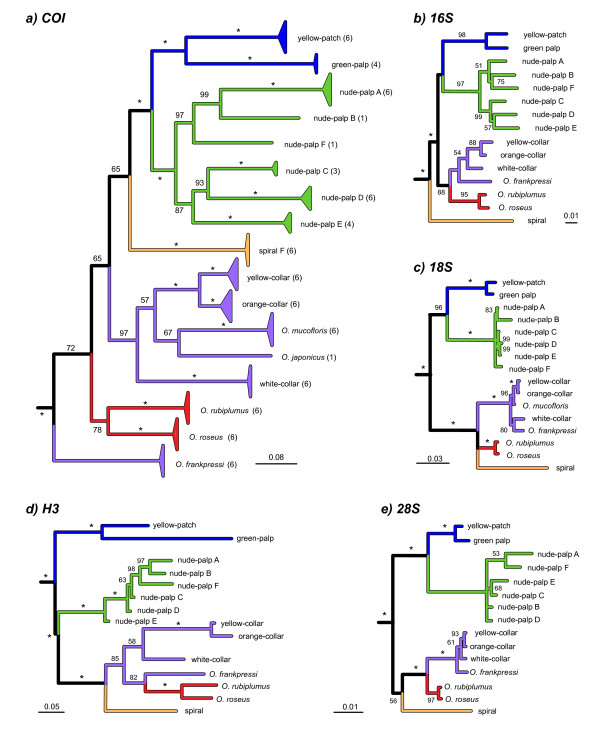
**Bayesian phylogenetic analyses of *Osedax *OTUs for portions of five genes: (*a*) mitochondrial *COI*; (*b*) *16S *rRNA; (*c*) *18S *rRNA; (*d*) Histone-*H3*; and (*e*)*28S *rRNA**. The black triangles in *a *represent the maximum depth and breadth of sequence diversity observed among multiple individuals (sample sizes in parentheses following OTU designations). The small numerals represent Bayesian Posterior Probabilities (BPP) expressed as percent, * = 100.

We initially conducted separate phylogenetic analyses for each gene. Altogether 83 *COI *sequences from *Osedax *clustered into 17 evolutionary lineages (Figure 2a). Multiple *COI *haplotypes were included, when possible, to represent the sequence divergence among (*D*) versus that found within (*π*) each lineage (Table 3). Only one *O. japonicus *sequence was available from GenBank, and to date we have collected only single individuals of *O*. nude-palp-F and *O*. nude-palp-B. The mean pairwise *D *values among the *Osedax *OTUs ranged from 8.4 to 24.3%. The smallest pairwise *D *value, obtained for *O*. yellow-collar versus *O*. orange-collar, was an order-of-magnitude greater than the largest *π *value observed within these OTUs (0.82%). *COI *transitions began to saturate after about 12% divergence, but transversions were not saturated and many of them resulted in amino acid substitutions (Table 2).

**Table 3:** *COI *sequence divergence (K2P corrected) within (*π *in *italics *on diagonal) and among (*D *on lower left) the *Osedax *OTUs.

	OTU	1	2	3	4	5	6	7	8	9	10	11	12	13	14	15	16	17
1	*O. rubiplumus*	0.0051																
2	*O. frankpressi*	0.1934	0.0034															
3	*O. roseus*	0.1573	0.1836	0.0047														
4	*O*. white-collar	0.1957	0.1985	0.2048	0.0017													
5	*O*. pinnules	0.2136	0.2131	0.2159	0.2350	0.0070												
6	*O*. green-palp	0.1795	0.1869	0.1859	0.2124	0.1952	0.0020											
7	*O*. yellow-collar	0.1896	0.1963	0.1831	0.1870	0.1897	0.2073	0.0048										
8	*O*. orange-collar	0.1838	0.2018	0.1844	0.1821	0.2018	0.2042	0.0843	0.0078									
9	*O*. spiral	0.2028	0.2069	0.2017	0.2148	0.2016	0.2086	0.1999	0.1990	0.0019								
10	*O*. nude-palp-A	0.2025	0.1929	0.2034	0.2354	0.2091	0.1879	0.2013	0.2016	0.2001	0.0082							
11	*O*. nude-palp-B	0.2028	0.1919	0.1902	0.2216	0.2181	0.1996	0.2166	0.2298	0.1817	0.1674	0.0000						
12	*O*. nude-palp-C	0.1899	0.1927	0.2001	0.2312	0.2057	0.2039	0.2188	0.1964	0.1966	0.1707	0.1593	0.0060					
13	*O*. nude-palp-D	0.1950	0.2204	0.1971	0.2367	0.2140	0.2054	0.2339	0.2191	0.2063	0.1956	0.1888	0.1643	0.0141				
14	*O*. nude-palp-E	0.2110	0.2250	0.2279	0.2087	0.2029	0.2228	0.2073	0.2176	0.1984	0.1926	0.1982	0.1713	0.1880	0.0072			
15	*O*. nude-palp-F	0.1781	0.2100	0.1909	0.1933	0.2072	0.1948	0.2084	0.2114	0.1919	0.1823	0.1643	0.1626	0.1822	0.1723	-		
16	*O. mucofloris*	0.2341	0.2106	0.2063	0.2138	0.2241	0.2432	0.1855	0.1856	0.2001	0.2075	0.2305	0.2061	0.2362	0.1950	0.2355	0.0060	
17	*O. japonicus*	0.2064	0.2125	0.1934	0.1788	0.2082	0.2145	0.1602	0.1426	0.2156	0.2215	0.2095	0.2307	0.2431	0.2378	0.2203	0.1726	-

Four additional genes revealed concordant phylogenetic differences among the *Osedax *OTUs (Figures 2b-e). The *16S*, *28S*, and *H3 *sequences differed among the 15 Monterey Bay OTUs, but sequences were not available for *O. japonicus *and *O. mucofloris*. Although their *18S *sequences were identical, *O*. yellow-collar and *O*. orange-collar differed from all the other Monterey OTUs and from *O. mucofloris*. The nodes leading to *O*. spiral and *O. frankpressi *were not stable, but all five gene-trees were broadly congruent in their topologies. Incongruence length difference (ILD) tests of homogeneity revealed that four of the five gene partitions were not significantly in conflict (*P *range: 0.119 - 1.00). Only the *H3 *tree was incongruent with respect to the *16S *and *18S *rRNA trees (*P *= 0.03 and 0.02, respectively). This problem resulted because *H3 *provided weak resolution among *Osedax *species that clustered together on a long branch relative to the outgroup *S. brattstromi*. ILD tests of homogeneity between *H3 *and the other partitions without the outgroup eliminated all remaining incongruence (*P *range: 0.125 - 1.00).

**Figure 3:**
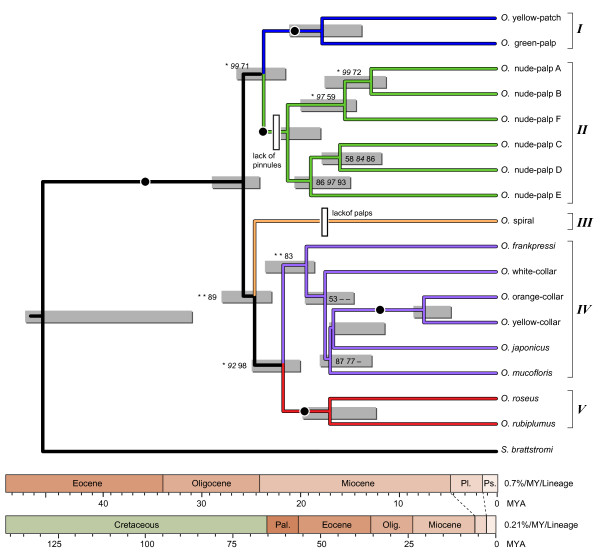
**Phylogenetic relationships among *Osedax *species based on concatenated sequences from two protein-coding genes (*COI *and H3) and three ribosomal RNA genes (*16S*, *18S*, and *28S*)**. Roman numerals at the right-hand margin delineate five *Osedax *species-groups. Three methods were used to denote the support for internal nodes: Bayesian posterior probabilities (BPP), maximum parsimony (MP) jackknife, and RAxML bootstrap values. If all three methods produced values ≥ 95%, the node is marked with a large black dot. Where support values differ, the BPP, RAxML (*italics*) and MP values are shown in order, and asterisks (_*_) equal 100%. Nodes that were not recovered with RAxML or MP analyses are indicated by a dash. Support values ≤ 50 are not shown. Based on most parsimonious reconstructions, the white rectangles mark the loss of palps in *O*. spiral and the loss of pinnules for the nude-palp species group.

Both the individual gene trees and the combined analysis involving concatenated sequences from all five genes identified several well-supported *Osedax *clades (Roman numerals *I - V*, Figure 3). Although limited sequence information was available for *O. mucofloris *(*COI *and *18S*) and *O. japonicus *(*COI*), they fell firmly within clade *IV*. *O. frankpressi *was also well supported as a member of clade *IV *in the combined analysis, but its position varied in the *COI *tree. Estimates of the age of *Osedax *depended on assumptions about rates of nucleotide substitution for mitochondrial *COI*. Mitochondrial *COI *divergence (*D*) between cognate species of shallow-water marine invertebrates isolated across the Isthmus of Panama grows at a rate of about 1.4% per MY [13]; so, the substitution rate (*r*_1_) equals *D*/2 or 0.7% per lineage per My. Assuming *r*_1 _= 0.7%, *Osedax *would have split from its monoliferan relatives about 45 MYA (95% HPD bounds: 31 - 47 Mya) (Figure 3). Time (*T*) to the most recent common ancestor for the *Osedax *would be 24 - 29 MY. Alternatively, assuming a slower substitution rate (*r*_2 _= 0.21% per lineage per MY) estimated for deep-sea hydrothermal vent annelids [14], *Osedax *would have split from monoliferans about 130 MYA (95% HPD bounds: 104 - 160 Mya). *T *for *Osedax *would be 81 - 97 My.

## Discussion

### Species diversity

Genetic and morphological differences among five previously named *Osedax *species provide a useful reference frame for assessing levels of divergence among the twelve undescribed OTUs considered in this study. *Osedax rubiplumus*, *O. frankpressi *and *O. roseus *live together on whale carcasses at depths greater than 1,000 m in Monterey Bay, CA (Figure 1; Table 1). To date, we have found no evidence for interbreeding among them. For example, an examination of 116 male *Osedax *sampled from the tubes of 77 *O. rubiplumus *females found no cases of foreign males in the female's tubes, despite the presence of *O. roseus *and *O. frankpressi *on the same carcass at 1820 m depth. Also including *O. mucofloris *from Sweden and *O. japonicus *from Japan, the mean sequence divergence (*D*) for mitochondrial *COI *between pairs of the named species was 19.6% (range: 15.7 to 23.4%; Table 2). Correspondingly, the mean pairwise *D *among the undescribed OTUs was 19.9% (range: 8.4 to 23.7%). The smallest value (8.4% between OTUs *O*. yellow-collar and *O*. orange-collar) was an order-of-magnitude greater than the largest divergence observed within any of these named or undescribed OTUs (*π *= 0.8% for *O*. nude-palp-A). These *π *values probably are underestimates, however, because each was obtained from a single locality. Isolation-by-distance and population subdivision across oceanic barriers are expected to increase *π *within broadly distributed species; however, *π *rarely exceeds 1 - 2%, unless other factors are involved. Global-scale phylogeographic surveys of *COI *sequence diversity have estimated *π *values less than 1% within named species of deep-sea hydrothermal vent annelids, mollusks and crustaceans, whereas *D *values typically are greater than 4% among species [14-22]. Nonetheless, odd cases of accelerated *COI *substitution rates have been reported. Sex-biased mitochondrial transmission and heteroplasmy are associated with accelerated divergence in some bivalve mollusks [23], but no evidence exists for this phenomenon in annelids, and we have found no differences in the distribution of mitochondrial haplotypes between males and females for *O. rubiplumus *[12]. High mitochondrial divergence rates have been reported for some marine and freshwater animals [24-26], but for the vast majority of cases mitochondrial *COI *is relatively conservative in its mutation rate within and among species. It is precisely the tendency of *COI *to discriminate clearly among the named species in many invertebrate taxa that has made this gene a common reference tool for DNA barcoding and molecular taxonomy [27,28].

Although numerous species concepts have been debated over the years [29,30], genealogical concordance across molecular and morphological characters provides a reliable indicator of longstanding evolutionary independence and consequently provides an operational criterion for species recognition [31]. Our confidence that the 12 presently unnamed OTUs from Monterey Bay represent distinct evolutionary lineages and warrant further consideration for naming as species is bolstered by morphological differences and concordant divergence observed across multiple gene loci. Only *18S *rRNA failed to distinguish between members of the closest pair of OTUs, *O*. yellow-collar and *O*. orange-collar. This highly conservative gene barely varies across the bivalve genus *Bathymodiolus*, globally widespread and diverse deep-sea mussels [32], or across a diverse clade siboglinid annelids, the vestimentiferans [9]. Consequently the *18S *differences reported for these *Osedax *OTUs are substantive. All the Monterey Bay OTUs also differed in their *16S, 18S, 28S *and *H3 *gene sequences. Phylogenetic trees generated independently from each of these genes clustered the OTUs in essentially similar ways (Figure 2). The combined analysis involving concatenated sequences from the five genes clearly reveals the evidence for long-standing evolutionary independence among these lineages. Only single individuals presently represent two of these lineages, *O*. nude-palp-D and -F. Nonetheless, these individuals differed from one another and clustered phylogenetically in a concordant fashion for all five genes. Formal descriptions of the new taxa shall be treated in forthcoming publications, as we obtain the additional samples needed for morphological studies and museum vouchers. Failure to formally recognize such highly divergent evolutionary lineages as distinct species creates a risk of significantly underestimating biological diversity [33]. In the meantime, attempts to identify these and other boneworms will be aided by the present gene sequences, which have been deposited in public databases including GenBank (Table 4) and the Barcode of Life Data System [34].

**Table 4:** GenBank accession numbers for the DNA sequences used in this study.

Taxa	*COI*	16S	18S	28S	H3
*O. rubiplumus*	EU223307--08, EU223298--99, DQ996618, 20	FJ347656	FJ347681	FJ347671	FJ347704
*O. frankpressi*	AY586495, EU223314, DQ996621, FJ347605--07	FJ347658	FJ347682	FJ347674	FJ347705
*O. mucofloris*	AY827562-568		AY941263		
*O. japonicus*	AB259569				
*O. roseus*	EU164760--61, EU032469--70, FJ347607-08	FJ347657	FJ347683	FJ347670	FJ347709
*O*. spiral	DQ996622--24, FJ347636--38	FJ347647	FJ347693	FJ347676	FJ347703
*O*. yellow-collar	DQ996629, 32--33, EU223332--33, EU223335	FJ347660	FJ347689	FJ347672	FJ347706
*O*. orange-collar	EU223340--41, EU223354, FJ347627-29	FJ347661	FJ347690	FJ347673	FJ347707
*O*. nude-palp-A	EU223356--58, FJ347622--24	FJ347653	FJ347687	FJ347662	FJ347702
*O*. nude-palp-B	EU236218	FJ347652	FJ347686	FJ347665	FJ347701
*O*. nude-palp-C	EU267675--76, J347625--26	FJ347650	FJ347688	FJ347666	FJ347710
*O*. nude-palp-D	FJ347630--31	FJ347649	FJ347691	FJ347667	FJ347708
*O*. nude-palp-E	FJ347632--35	FJ347648	FJ347692	FJ347664	FJ347700
*O*. nude-palp-F	FJ347643	FJ347651	FJ347695	FJ347663	FJ347699
*O*. white-collar	FJ347610--15	FJ347659	FJ347684	FJ347675	FJ347712
*O*. yellow-patch	FJ347616--21	FJ347654	FJ347685	FJ347668	FJ347698
*O*. green-palp	FJ347639--42	FJ347655	FJ347694	FJ347669	FJ347711
*S. brattstromi*	FJ347645	FJ347680	FJ347677	FJ347697	FJ347645

### Phylogeny

Individual gene trees (Figure 2) and the combined phylogenetic analysis (Figure 3) identified several well-supported groupings within *Osedax *(clades *I - V*). *Osedax *spiral (clade *III*) stands alone as the most atypical of these worms. Its oviduct does not extend beyond the trunk, and it lacks the vascularized anterior palps that characterize all other *Osedax *(Figure 1g). Unlike all other *Osedax, O*. spiral is a late successional species which lives at the sediment interface and produces long fibrous *roots *that penetrate the anoxic (black and sulfidic) sediments to exploit buried fragments of bone [5]. The lack of palps in *O*. spiral probably represents a character loss under a most parsimonious reconstruction, because all other siboglinids bear an anterior crown composed of one or more palps. The nude-palp OTUs (clade *II*) differ because their palps do not bear the lateral pinnules seen in the other *Osedax *clades (Figures 1i and 1j). Lack of pinnules may represent a character loss, but supporting evidence regarding the homology and distribution of pinnules in other siboglinids is uncertain. Monoliferans have two or more palps, with numerous pinnules in the case of Vestimentifera, but pinnules are absent in *Sclerolinum *and in some frenulates [10,35].

The remaining *Osedax *clades (*I, IV *and *V*) bear four palps with numerous pinnules that give the crown a feathery appearance (e.g., Figure 1a). The two members of clade *V *have long branched roots that are green in color (Figures 1e-f) and palps that are bright red with outwardly facing pinnules. Clades *II *and *V *share robust lobate roots. The two members of clade (*I*) have relatively short trunks and palps (Figure 1h), but they have not been found in great numbers because they are small and may have been overlooked in earlier samples. Members of clade *IV*, which have red, pink or even white crowns (Figures 1a-d), were recovered from depths of 1,020 m or less, excepting *O. frankpressi*, which has not been found shallower than 1,800 m. Occupation of shallow habitats might be a derived condition for these members of clade *IV*, though support for a *shallow *clade was weak (Figure 3). Addition of comparative sequence data from the other shallowmembers of this clade, *O. japonicus *and *O. mucofloris*, might help to strengthen this relationship (only *18S *and *COI *data are available on GenBank for *O. mucofloris *and only *COI *for *O. japonicus*). Otherwise, no clear evolutionary pattern of depth utilization is apparent among the major *Osedax *clades. Several of these OTUs were sampled from a single depth, others were sampled across relatively narrow depth ranges (300 - 600 m for *O*. yellow-patch and *O*. orange-collar), and some were sampled across broad depth ranges (1,000 m for *O. frankpressi*, and *O. rubiplumus *and 1,200 m for *O. roseus*).

### Age of Osedax

For now, we are unable to confidently delineate a timeframe during which *Osedax *split from its monoliferan relatives or the age (*T*) of the most recent common ancestor for this unusual genus. Present evidence indicates that *Osedax *species live primarily on organic compounds extracted directly from sunken bones. Their Oceanospirillales symbionts are capable of growing on collagen and cholesterol as primary carbon sources [6]. Video evidence suggests that *O. japonicus *also grows on spermaceti, a wax found in the head of sperm whales [3]. There are arguments to suggest that *Osedax *may not be nutritionally restricted to living on whale-falls. Experimental deployments of cow bones and observations of sunken possible pig bones reveal that *Osedax *can grow and reproduce on a range of mammalian tissues including those from terrestrial quadrupeds [7,36]. So, it may be unwarranted to associate the evolution of these bone-eating worms with the origin and spread of oceanic whales, as previously suggested by Rouse et al. [1]. Nonetheless, one of the scenarios that we have considered here is consistent with that hypothesis. If we assumed a divergence rate (*d *= 1.4% per MY) calibrated for mitochondrial genes from shallow-water marine invertebrates [13], and applied this rate (*r*_1 _= *d/*2 = 0.70%/lineage/My) to *COI *divergence, we estimated that *Osedax *split from its monoliferan relatives about 45 Mya, possibly coincident with the origins of large archeocete cetaceans during the Eocene [37]. According to this scenario, the most recent common ancestor for the *Osedax *sampled to date would have lived about 26 MYA, during the Late Oligocene and roughly coincident with the diversification of modern cetaceans [38].

Alternatively, we can assume a slower substitution rate (*r*_1 _= 0.21%/lineage/My) calibrated from *COI *divergence in deep-sea annelids, including Vestimentifera [14], as was used by Rouse et al. [1] for estimating the origin of *Osedax *when only *O. rubiplumus *and *O. frankpressi *were known. Under this rate then *Osedax *would appear to be much older than previously hypothesized [1]. This result is not surprising given the larger diversity of *Osedax *shown here. Accordingly, *Osedax *split from its monoliferan relatives during the Cretaceous, and the most recent common ancestor for the genus would have lived during the Late Cretaceous. Perhaps the calcified cartilage and bones from a variety of large Cretaceous vertebrates supported these worms -- e.g., mosasaurs, plesiosaurs, turtles, and possibly chondrichthyans and teleosts [39-42]. Fossilized snails and bivalves were recently found with plesiosaur bones; so the sunken carcasses of these large marine reptiles appear to be capable of supporting communities much like those found on modern whale-falls [43]. Nevertheless, this scenario is problematic, because the major *Osedax *clades would have diversified around the Cretaceous-Tertiary (K/T) boundary, after the extinction of most large-bodied reptilians [44]. Although dyrosaurid crocodylomorphs survived the K/T event, they were confined to relatively shallow coastal environments [45] and probably would not have supported *Osedax*. Large turtles and chondrichthyans also survived the K/T boundary [42], and large teleosts appeared again during the early Paleocene [46]. It is unknown whether *Osedax *can exploit these resources; so, arguably a 20 MY gap may have existed during the Paleocene when there would have been little in the way of large vertebrate remains for *Osedax*. Another problem with this scenario is the concern that nucleotide substitution rates may be slower in the deep-sea vent annelids used to obtain the *r*_2 _= 0.21% calibration rate [9].

## Conclusion

The present phylogenetic evidence based on DNA sequences from multiple independent genes provides a solid foundation for future discoveries and taxonomic descriptions of *Osedax *species. However, our efforts to estimate evolutionary ages for the diversification of this unusual group of worms only allowed the erection of new hypotheses that could be tested with independent evidence from the fossil record. Soft-bodied invertebrates like *Osedax *do not often leave convincing fossils, but these worms might leave traces of their activity by the distinctive holes they bore into bones. To date, we have found no other animals that create similar borings in bones. Consequently, we have distributed whalebones containing *Osedax *to several paleontologists who are also examining the taphonomy of fossilized bones from plesiosaurs and cetaceans. It is to be hoped that these efforts will help us to narrow the age of this remarkable genus of bone-eating worms.

## Materials and methods

### Specimens

Locations of the Monterey Bay whale-falls, except whale-634, are provided elsewhere [5]. Whale-634 is the carcass of a juvenile gray whale that was sunk on 5 October 2004 at a depth of 633 m at 36.802°N and 122.994°W. We used the remotely operated vehicles, ROV *Tiburon *and ROV *Ventana*, operated by the Monterey Bay Aquarium Research Institute (MBARI) to collect *Osedax*-inhabited bones from five whale-fall localities (Table 1). Bones were transported to the surface in closed insulated containers and stored temporarily in cold (4°C) filtered seawater. Worms were dissected from the bones and photographed. Then a palp tip was removed and stored in 95% ethanol or frozen immediately at -80°C. The remainder of each specimen was preserved for anatomical studies and taxonomic descriptions. Voucher specimens were lodged in Scripps Institution of Oceanography Benthic Invertebrate Collection (catalogue numbers in Table 1). Other specimens will be distributed to other Museums upon their formal description (Rouse, in progress). For the present purpose, we list the approximate sizes (trunk plus crown length) and several morphological characteristics of each OTU (Table 1).

Published DNA sequences from *Osedax mucofloris *(*18S *rRNA and *COI*) and *O. japonicus *(*COI*) were recovered from GenBank [2]. A previous phylogenetic analyses [1] placed *Osedax *in a clade that also includes the Monolifera, which includes *Sclerolinum *and vestimentiferan tubeworms [10]. The frenulates, a diverse group of slender chemosynthetic worms are basal to the monoliferans and *Osedax *[10,47]. Ongoing studies of siboglinid phylogeny revealed that *Sclerolinum *is presently our best choice as outgroup for this study of *Osedax *phylogeny. The vestimentiferan *Lamellibrachia columna *was also examined, and its substitution as outgroup did not substantively alter the tree topologies for the ingroup. Other vestimentiferans were not considered, however, because incomplete sequence data are available and because independent evidence from several genes suggests that rates of nucleotide substitution may have slowed down in these deep-sea worms [9,14,48]. Consequently, we have used DNA sequences from the monoliferan *Sclerolinum brattstromi*, collected near Bergen, Norway. GenBank accession numbers for all the DNA sequences used in this study are listed in Table 4.

### DNA methods

Total DNA was extracted using the DNeasy kit (Qiagen, Valencia, CA, USA) according to manufacturer's instructions. We used primers that amplified approximately 1200 bp of *COI *[49], approximately500 bp of *16S *rRNA [50], approximately 1000 bp of *28S *rRNA [51], approximately 1800 bp of *18S *rRNA [52], and approximately 370 bp of *H3 *[53]. Amplification reactions with AmpliTaq Gold (Applied Biosystems Inc., Foster City, CA, USA) were conducted in a GeneAmp 9700 thermal cycler (Applied Biosystems Inc., Carlsbad, CA, USA) with the following parameters: 95°C/10 min, 35× (94°C/1 min, 55°C/1 min, 72°C/1 min), and 72°C/7 min. If available, at least six individuals of each species were sequenced for each locus. PCR products were diluted in 50 μl sterile water and cleaned with Multiscreen HTS PCR 96 filter plates (Millipore Corp., Billerica, MA, USA). The products were sequenced bidirectionally with the same primers on an ABI 3100 sequencer using BigDye terminator v.3.1 chemistry (Applied Biosystems Inc., Foster City, CA, USA).

### Phylogenetic analyses

Sequences were assembled using CodonCode Aligner v. 2.06 (CodonCode Corporation, Dedham, MA, USA), aligned using Muscle [54] and edited by eye using Maclade v. 4.08 [55]. We used MrModelTest [56] and the Akaike information criterion [57] to determine appropriate evolutionary models for each gene (Table 2). *COI *and *H3 *were partitioned by codon position, and parameters were estimated separately for each position. RNA secondary structures were predicted with GeneBee and used to partition stems and loops in *16S*, *18S*, and *28S *sequences. The doublet model was used for RNA stems and a standard 4 × 4 nucleotide model was used for RNA loops. The number of indel haplotypes for rRNA sequences (total number of indels, number after excluding overlapping indels, and average length of indels) were estimated with DNAsp v. 4.90.1 [58] using the diallelic model. Gaps in the RNA sequences were treated as a fifth character-state in subsequent Bayesian phylogenetic analyses and as missing data in parsimony and maximum likelihood (ML) analyses. The program DAMBE [59] was used to examine saturation of the mitochondrial *COI *sequences for the *Osedax *OTUs and outgroup taxa.

First, each gene was analyzed separately using MrBayes v. 3.1.2 [60,61]. Bayesian analyses were run as six chains for 5·10^6 ^generations. Print and sample frequencies were 1,000 generations, and the burn-in was the first 100 samples. We used AWTY [62] to assess whether analyses reached convergence and FigTree v. 1.1.2 [63] to display the resulting trees. We then used the incongruence length difference (ILD) function implemented in Paup* v. 4.0 [64] to assess congruence of the tree topologies produced by the individual gene partitions. ILD tests were conducted both with and without the outgroup taxa. The ILD partition homogeneity test was run for 1,000 replicates with 10 random additions of gene sequences.

A combined analysis was conducted with concatenated sequences from the five genes. If available, multiple individuals of each OTU were sequenced for each gene; however, the concatenated multilocus sequences used in the phylogenetic analyses were obtained from a single representative individual for each OTU. The five gene regions were partitioned separately according to the previously determined model parameters. Bayesian phylogenetic analyses were then conducted with MrBayes v. 3.1.2. Maximum parsimony analysis of the combined data set was performed with Paup* v. 4.0 [64] using an equally weighted character matrix, heuristic searches using the tree-bisection-reconnection branch-swapping algorithm, and 100 random addition replicates. The resulting shortest tree included 3481 steps. A parsimony jackknife analysis (with 37% deletion) was run for 100 iterations with the same settings as the parsimony search. ML analysis was conducted using RAxML 7.0.4 (with bootstrapping) using GTR+I+G as the model for each partition on combined data. RAxML analyses were performed with the CIPRES cluster at the San Diego Supercomputer Center.

### Relaxed molecular clock

A Bayesian, MCMC method implemented in Beast v. 1.4.8 [65] was used to estimate the evolutionary ages of internal nodes in the tree topology derived from the combined phylogenetic analysis. Estimates of the time to most recent common ancestor (*T*) were based on two calibrations nucleotide substitution rates for mitochondrial *COI*. Substitution rates (*r*) were estimated as percentage per lineage per million years (my) so they equal one-half the divergence per unit of time (*T*) between taxa (*r *= 100 × *D*/2*T*). First, we assumed a conventional substitution rate, *r*_1 _= 0.7%, based on *D *= 1.4% per my pairwise divergence rate commonly cited for shallow water marine invertebrates that were isolated by the emergence of the Isthmus of Panama [13]. Second, we used a slower rate, *r*_2 _= 0.21%, previously calibrated from a vicariant event that split cognate-species of deep-sea hydrothermal vent annelids between the East Pacific Rise and the northeastern Pacific ridge system about 28.5 myA [14]. Calibrations were not available for the other genes.

We used a relaxed, uncorrelated, lognormal molecular clock with a general time reversible (GTR) substitution model that was unlinked across codon positions. Initial MCMC test runs consisted of 10 million generations to optimize the scale factors of the prior function. Three independent MCMC chains were run for 100 million generations, sampled every 1000 generations. Results were visualized in and FigTree v. 1.1.2 and Tracer v. 1.4 [66].

## Abbreviations

16S: mitochondrial large subunit ribosomal RNA; 18S: nuclear small subunit subunit ribosomal RNA; 28S: nuclear large subunit ribosomal RNA; COI: cytochrome oxidase subunit I; GC: guanine-cytosine; GTR: general time reversible; H3: Histone 3; K/T: Cretaceous-Tertiary; MCMC: Monte Carlo Markov chain; MYA: million (10^6^) years ago; OTUs: operational taxonomic units; ROV: remotely operated vehicle.

## Authors' contributions

RCV conceived of the project, directed the research, and composed much of the manuscript. SBJ obtained the DNA sequences and conducted most of the phylogenetic analyses. GWR obtained the images of *Osedax *OTUs, conducted morphological examinations, and contributed to the phylogenetic analyses and writing of the manuscript.
